# Network analysis of anxiety and depressive symptoms during the COVID-19 pandemic in older adults in the United Kingdom

**DOI:** 10.1038/s41598-024-58256-8

**Published:** 2024-04-02

**Authors:** Cristian Ramos-Vera, Angel García O’Diana, Miguel Basauri-Delgado, Yaquelin E. Calizaya-Milla, Jacksaint Saintila

**Affiliations:** 1 Área de Investigación, Universidad César Vallejo, Lima, Peru; 2 Doctorando en Psicología, Universidad Femenina del Sagrado Corazón (UNIFE), Lima, Peru; 3 Facultad de Ciencias de la Salud, Universidad Peruana Unión, Chosica, Lima 15, Perú; 4 Escuela de Medicina Humana, Facultad de Ciencias de la Salud, Universidad Señor de Sipán, Chiclayo, Peru

**Keywords:** Depression, Anxiety, Network analysis, Cross-lagged panel network, Older adult, Psychiatric disorders, Public health

## Abstract

The health crisis caused by COVID-19 in the United Kingdom and the confinement measures that were subsequently implemented had unprecedented effects on the mental health of older adults, leading to the emergence and exacerbation of different comorbid symptoms including depression and anxiety. This study examined and compared depression and anxiety symptom networks in two specific quarantine periods (June–July and November–December) in the older adult population in the United Kingdom. We used the database of the English Longitudinal Study of Aging COVID-19 Substudy, consisting of 5797 participants in the first stage (54% women) and 6512 participants in the second stage (56% women), all over 50 years of age. The symptoms with the highest centrality in both times were: “Nervousness (A1)” and “Inability to relax (A4)” in expected influence and predictability, and “depressed mood (D1”; bridging expected influence). The latter measure along with "Irritability (A6)" overlapped in both depression and anxiety clusters in both networks. In addition, a the cross-lagged panel network model was examined in which a more significant influence on the direction of the symptom "Nervousness (A1)" by the depressive symptoms of "Anhedonia (D6)", "Hopelessness (D7)", and "Sleep problems (D3)" was observed; the latter measure has the highest predictive capability of the network. The results report which symptoms had a higher degree of centrality and transdiagnostic overlap in the cross-sectional networks (invariants) and the cross-lagged panel network model of anxious and depressive symptomatology.

## Introduction

The COVID-19 pandemic has had a significant impact on the United Kingdom (UK), with cases being reported since the end of January. However, the British government delayed the start of quarantine until March 2020 because they implemented other strategies, such as herd immunity^1^. Between September and December of the same year, there was an increase in the number of COVID-19 cases due to a new variant of the virus; this led the UK government, along with other European countries and countries around the world, to implement a second COVID-19 containment measure^2,3^. The periods of confinement had a negative impact on people's mental health, as they favored a greater occurrence of symptoms related to anxiety and depression^4–8^. Older adults experience a greater fear of contracting COVID-19 due to their vulnerability as a population^9^; which is intensified by the context of uncertainty and negative news related to COVID-19^10,11^. As individuals age, it is common for them to experience a reduction in mobility, the onset of chronic or degenerative diseases, and increased frailty. This often requires the assistance of others and can trigger negative emotional states^12,13^.

At the beginning of the COVID-19 confinement in the UK, a survey revealed that the mental health of the population was most commonly affected by depression and generalized anxiety^5^. Similarly, various studies indicate a strong correlation between the two variables due to shared symptoms, such as fear, loss of control, and difficulty maintaining concentration^14,15^. Similarly, generalized anxiety is a continuous phenomenon that is often comorbid with depression; this can lead to increased functional disability, worse prognosis, and the persistence of other illnesses^7^. However, network analysis can provide a systematic understanding of the interaction between different psychological constructs, including anxiety and depression symptoms^16^. Furthermore, it is possible to draw inferences about comorbidity of symptoms by analyzing the most central items or symptoms in the network. These central symptoms can activate other symptoms and are crucial for maintaining the stability of the psychopathological system^17,18^.

Various studies have utilized network analysis to comprehend the most significant symptoms of anxiety and depression amid the COVID-19 pandemic. For example, in a study of 3946 older adults in Hong Kong during the initial period of confinement, Jin et al. (19) found that sad mood and guilt were the most central symptoms in a network of depressive symptoms. Zhang et al. (19) found that nervousness and excessive worry were the most interconnected indicators in a network that included symptoms of anxiety and depression among 1302 older adults in China. Li et al. (19) analyzed a network of multiple clinical symptoms during the COVID-19 outbreak in the same country with 1063 older adults; the symptoms included pain, fatigue, post-traumatic stress, anxiety, and depression, among others; the study found that depression was the most influential node in the network, followed by anxiety. According to Liu et al. 28, indicators of depressed affect and nervousness were central to a network of symptoms of anxiety and depression during the confinement state. In a separate study, Chinese older adults were evaluated during the second period of COVID-19 confinement to review the network of anxiety and depression symptoms. The study reported that the symptom of restlessness was strongly connected to other indicators in the network^19^.

In a longitudinal network analysis study with adults from the United Kingdom, two distinct communities for anxiety and depression indicators were identified during the first month of the confinement state; however, for the second assessment, after 1 month, a grouping within a single community of anxious and depressive symptoms was observed^20^. Similarly, in their analysis of a longitudinal network of affective symptoms in older adults in Singapore, Yu and Mahendran^21^ found that the interconnectedness between anxiety and depression was greater during the period of COVID-19 confinement. Prior to social isolation, they recorded a more dispersed connection between affective symptoms.

However, network analysis allows the investigation of comorbid symptoms between disorders such as anxiety and depression, which facilitates the recognition of symptoms that may hinder the diagnostic process and the identification of the most appropriate treatment^22^; To date, most previous studies have used network analysis to understand and compare depressive and anxious symptomatology at different periods of the pandemic in adolescents, adults, and older adults in different countries, including the United Kingdom^19,23–25^. However, there is a gap in research with older adults utilizing the clique-percolation statistic to analyze a network with symptoms of depression and anxiety during two distinct periods of the pandemic (first and second waves). These studies are important because they enable us to identify symptoms that belong to multiple groups or clusters simultaneously, as well as to recognize comorbid symptoms that reinforce the co-occurrence of these emotional disorders^26,27^. Furthermore, this analysis also allows exploring the variables from a transdiagnostic perspective, as it refers that these symptoms can be identified in both diagnoses^28,29^, as reported by previous findings on the overlapping nature of these psychopathological symptomatologies^30,31^.

The purpose of this study was to compare the depression and anxiety symptom networks in two specific quarantine periods among older adults in the UK. The study aimed to reinforce the findings of clustering and centrality from a cross-lagged panel network model perspective. The study utilized the Clique Percolation Method (CPM) to investigate symptoms that may belong to multiple psychopathological communities or overlap with depressive and anxious symptomatology. We aimed to identify the most influential measures of network structure and topology that would provide a better understanding of the co-occurrence of symptoms in a national sample of older adults.

## Methods

### Participants

This study examined data from individuals over 50 years of age from the English Longitudinal Study of Aging (ELSA) COVID-19 Substudy (ELSA), a national survey that assessed aspects of physical and mental health in the United Kingdom during phases of rigorous quarantine due to COVID-19. The study collected data on the health impacts of COVID-19 in two phases: the first phase between June and July 2020 (Wave 1) and the second phase between November and December 2020 (Wave 2)^31^. In addition, anxiety and depression symptoms were assessed through online questionnaires and computer-assisted interviews via telephone. In the first wave, 7040 responses were collected, while in the second wave, 6794 responses were obtained. After discarding inaccurate or missing data, the final number of participants in the first period was 5797, and in the second period, there were 6512 older adults^31^. For additional information on the sampling method and survey technique, you can visit www.elsa-project.ac.uk.

### Ethical considerations

Ethical clearances for ELSA were obtained through the London Multicenter Research Ethics Committee (MREC/01/2/91)^32^. More details on the ethical approval of each phase of ELSA can be found at https://www.elsa-project.ac.uk/ethical-approval. The study, which involved only existing and anonymized data, was confirmed by the Research Ethics Committee of the Universidad Peruana Unión and did not require additional ethical clearance (Registration Number: 2023-CEUPeU-0014). The study was conducted in accordance with the Declaration of Helsinki. All ELSA participants gave informed consent before being included in the research.

### Instruments

*Center for Epidemiological Studies-Depression Scale (CES-D)* Depression was measured with the CES-D developed by Radloff^33^ in its 7-item version (e.g., “Much of the time during the past week: felt sad”) under a unidimensional model. The scale is based on responses to seven questions in a dichotomous format, where in this study the values "no" (1) and "yes" (2) were assigned. This allows the generation of a continuous measure that spans a range from 7 to 14, where higher scores indicate higher levels of depressive symptoms experienced during the last week.

*Generalized Anxiety Disorder scale (GAD-7)* To measure anxiety, the GAD-7 created by Spitzer et al.^34^ was used to detect the severity of generalized anxiety symptomatology according to DSM-V criteria over the last 2 weeks. It consists of 7 items (e.g., “Over the last 2 weeks: Feeling nervous, anxious, or on edge”) within a unidimensional model, with a Likert-type response mode ranging from 0 to 3 points (never, several days, half of the days and almost every day). The total scale has a minimum score of 0 and a maximum of 21.

### Data analysis

All data and analyses were processed with the R language in the R Studio GUI^35^, using the packages: qgraph^36^, igraph^37,38^, bootnet^39^, CliquePercolation^40^, networktools^41^, NetworkComparisonTest^42^ and lavaan^43^.

The network structure of partial correlations for both models was calculated with the bootnet package^39^, through the huge estimator^44,45^, a non-paranormal conversion of the data^46^ and the Rotation of Information Criterion (RIC), was used for model selection^47^.

The stability and accuracy of each network model for each time was calculated through 5000 nonparametric Bootstrap and person-dropping samples, where a coefficient of stability (CS) greater than > 0.5 indicates strong stability and interpretability^48^.

Communities were explored with the spinglass clustering algorithm^49–51^ through 500 spins at the two times.

Centrality was explored through the expected influence step 1 (EI1; which is the sum of the weights of the axes at directly related nodes) and step 2 (EI2; which is the sum of the weights of the axes at indirectly related nodes)^52^. Also included was the bridge expected influence step 1 (BEI1; sum of the weights of the axes connecting each node with the nodes of other directly related communities) and step 2 (BEI2; sum of the weights of the axes connecting each node with the nodes of other indirectly related communities)^41^.

Subsequently, an analysis of the overlapping communities was performed with the cpAlgorithm function in the CliquePercolation package^40^, percolated items were found through the 'weighted CFinder' method^53^; with a configuration of k = 4 cliques and an intensity of I = 0.08, for the cross-lagged panel network model.

Then, the networks of both times were compared with the NCT function of the NetworkComparisonTest package^42^, with the bonferroni-holm correction technique^54,55^ and 1000 permutations. The similarity of the networks is explored through the correlations of the adjacency matrices and their centrality indices, if the result is 1, the networks have a perfect linear relationship, which means that the networks have the same structure; if the correlation is 0, the networks have no detectable linear relationship, so they have no correspondence and if the correlation coefficient is − 1, the networks are exact opposites^56^.

Finally, a two-step cross-lagged panel network (CLPN) analysis was performed for both estimation and summary results. Measures of centrality in the form of in-prediction and out-prediction were also calculated^57^.

## Results

Table 1 shows the descriptive results at the item level, in addition to the expected influence and the final bridging expected influence scores. The item "Nervousness (A1)" obtained the highest scores at both times (T1; x = 1.49; SD = 0.77; T2; x = 1.57 SD = 0.83). The skewness and kurtosis values exhibit some outliers. Although, for the analyses performed, the confirmation or not of multivariate normality is not essential, the non-normal data transformation method was used to mitigate this particularity.

**Table 1:** Descriptive analysis, centralities, and predictability.

T	Item	x	SD	g1	g2	EI	BEI	R2
T1	A1	1.49	0.77	1.71	2.57	1.09	0.31	0.61
	A2	1.34	0.69	2.29	5.16	1.05	0.42	0.59
	A3	1.47	0.74	1.71	2.69	1.08	0.3	0.6
	A4	1.47	0.76	1.75	2.69	1.16	0.42	0.56
	A5	1.34	0.69	2.31	5.13	0.76	0.3	0.39
	A6	1.48	0.71	1.58	2.51	0.73	0.38	0.35
	A7	1.34	0.67	2.24	5.08	0.78	0.24	0.45
	D1	1.17	0.37	1.8	1.25	0.94	0.64	0.4
	D2	1.22	0.41	1.36	-0.16	0.88	0.5	0.4
	D3	1.44	0.5	0.23	-1.95	0.47	0.31	0.18
	D4	1.15	0.36	1.91	1.64	0.87	0.43	0.44
	D5	1.16	0.36	1.89	1.56	0.57	0.3	0.22
	D6	1.18	0.38	1.67	0.78	0.93	0.41	0.45
	D7	1.26	0.44	1.1	-0.8	0.81	0.42	0.34
T2	A1	1.57	0.83	1.5	1.65	1.06	0.38	0.61
	A2	1.44	0.76	1.84	2.92	1.09	0.41	0.62
	A3	1.56	0.8	1.52	1.9	1.1	0.32	0.62
	A4	1.53	0.8	1.58	1.96	1.13	0.37	0.56
	A5	1.35	0.71	2.24	4.6	0.76	0.28	0.4
	A6	1.53	0.76	1.51	2.02	0.75	0.43	0.37
	A7	1.38	0.71	2.07	4.04	0.84	0.4	0.47
	D1	1.19	0.39	1.6	0.57	0.93	0.67	0.4
	D2	1.25	0.43	1.15	-0.67	0.94	0.56	0.43
	D3	1.47	0.5	0.1	-1.99	0.49	0.34	0.2
	D4	1.19	0.39	1.61	0.6	0.83	0.46	0.41
	D5	1.18	0.38	1.69	0.87	0.57	0.35	0.23
	D6	1.21	0.41	1.4	-0.03	0.91	0.45	0.42
	D7	1.3	0.46	0.88	-1.22	0.8	0.44	0.37

*T* survey waves, *T1* wave 1, *T2* wave 2, *x* mean, *SD* standard deviation, *g1* skewness, *g2* kurtosis, *EI* expected influence, *BEI* bridge expected influence, *R2* predictability.

**Figure 1:**
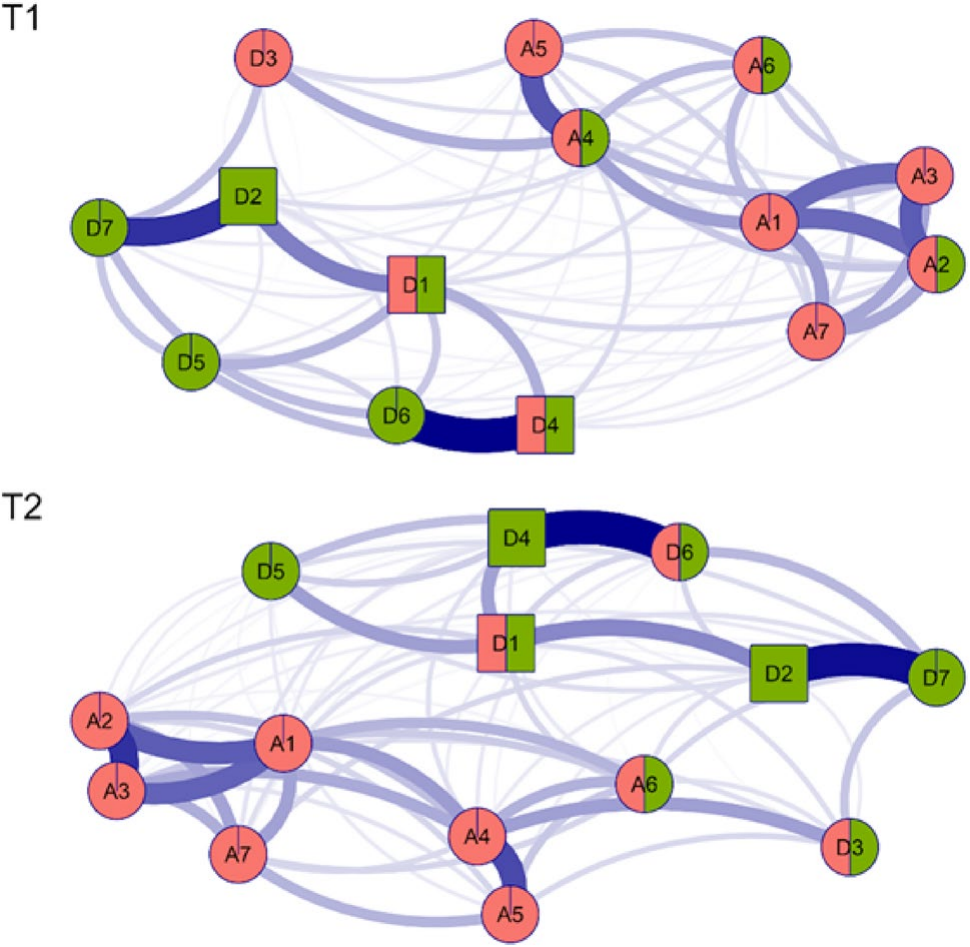
Network analysis of both waves (T1 and T2). *Note*: Red cluster: Anxiety; Green cluster: Depression. The percolated nodes are shown in 2 colors. The items with the highest centrality (BEI) sustained in both times have a square shape. Inverse items are marked with (R). A1: Nervousness, A2: Worry management, A3: Excessive worry, A4: Inability to relax, A5: Restlessness, A6: Irritable, A7: Fear, D1: Depressed, D2: Fatigue, D3: Restless sleep, D4: Happiness (R), D5: Loneliness, D6: anhedonia (R), D7: Hopelessness.

In the models of both networks (Fig. 1), positive relationships between nodes were identified, highlighting the relationship between "Anhedonia (D6)" and "Happiness” (D4; T1 r = 0.444; T2 r = 0.402), as well as the relationship between "Hopelessness (D7)" and "Fatigue” (D2; T1 r = 0.359; T2 r = 0.383) in both networks. In addition, at the second time, a higher partial correlation was observed between "Excessive worry (A3)" and "Lack of worry control” (A2; T2 r = 0.324). Correlation matrices for the two network models presented can be found in the Appendices section (Appendix 1 and 2). The clusters analyzed revealed a coherent partitioning, while the percolated communities highlighted that for the first time (T1), the items "Worry management (A2)", "Inability to relax (A4)", "Irritable (A6)", "Depressed mood (D1)" and "Happiness (D4)" presented an overlap in the anxiety and depression communities. In addition, in this same period, the item "Restless sleep (D3)" was associated with the anxiety community. On the other hand, at time 2 (T2), the items of "Irritability (A6)", "Depressed mood (D1)", "Restless sleep (D3)", and "Anhedonia (D6)" are overlapped by both communities. Finally, the coefficient of stability (CS) for the axes and strength at both times was CS = 0.75.

For T1, the item "Nervousness (A1)" had the highest predictability (r2 = 0.61), while "Restless sleep (D3)" had the lowest value (r2 = 0.18). Likewise, for T2, the item “Excessive worry (A3)” was the highest (r2 = 0.62) and "Restless sleep (D3)" was the lowest (r2 = 0.20).

Additionally, centrality indices (Table 1) showed that at time 1 (T1) the most central item was "Inability to relax” (A4; EI = 1.16) and the lowest was "Restless sleep” (D3; EI = 0.47); while in bridge centrality, the most central was "Depressed mood” (D1; IEP = 0.64) and the least central was fear (A7; IEP = 0.24). On the other hand, at time 2 (T2) the centralities differed for each step, at step one of the expected influences, the most central item was “Inability to relax” (A4; IE = 1.13), while at step two it was “Excessive worry” (A3; IE = 1.10). For both centralities, the lowest value was “Restless sleep” (D3; IE = 0.49). Likewise, regarding the bridge expected influence, the item with the highest value was “Depressed mood” (D1; IEP = 0.67), while the lowest was “Restlessness” (A5; IEP = 0.28).

**Figure 2:**
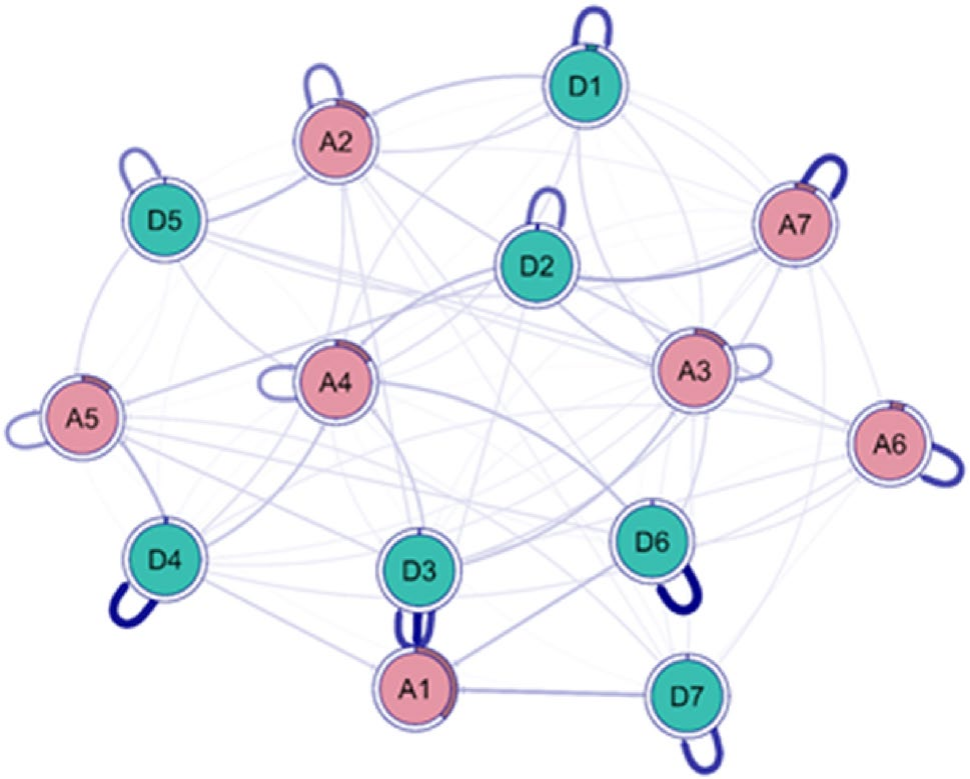
CLPN of the anxiety and depression model. *Note*: The axes are refined according to the significance of the relationships. The temporal autoregressions of the nodes are shown. A1: Nervousness, A2: Worry management, A3: Worry, A4: Inability to relax, A5: Restlessness, A6: Irritable, A7: Fear, D1: Depressed, D2: Fatigue, D3: Restless sleep, D4: Happiness (R), D5: Loneliness, D6: Anhedonia (R), D7: Hopelessness.

Figure 2 shows the graph resulting from the CLPN analysis, in which the autoregressions can be observed, as well as the standardized regressions between the nodes. For the first case, the highest scoring autoregressions were "Anhedonia” (D6; β = 0.445) and "Happiness” (D4; β = 0.414), the regression matrix can be found in Appendix 4. On the other hand, the highest scoring inter-node regressions were from "Restless sleep" to "Nervousness (D3 → A1; β = 0.421)" and from "Fatigue" to "Fear (D2 → A7; β = 0.154)". As for the centralities (Appendix 3), the node with the highest in-prediction was "Nervousness" (A1; inPred = 0.38), meaning that this node was the most affected by the others in the model analyzed; while the node with the highest out-prediction was "Restless sleep" (D3; outPred = 0.21), i.e., this symptom was the one that most affected the others in the model.

The network comparison test between times T1 and T2 (Table 2) showed statistical significance in the differences only between network centrality indices (*p* < 0.05). Likewise, the correlation between adjacency matrices showed linearity (rho = 0.89; *p* < 0.001), as well as in the centrality indices. Additionally, at the item level, both axis strengths and centralities reported no significant differences.

**Table 2:** Network invariance analysis and expected influence invariance.

Network models	Network/central invariance		Correlation of adjacency matrices	
	M *p* value	S *p* value	rho	*P* value
T1 T2	0.21	0.03	0.89	< .001
Centrality	Rho	*p* value		
EI	0.96	< .001		
BEI	0.78	< .001		

*M* inter-network invariance, *S* invariance between centrality strength, *rho* Spearman's coefficient, *EI1* expected influence, *BEI* bridge expected influence.

*p* value *< .01; **< .05; *** < .001.

## Discussion

The current study's main objective was to examine and compare depression and anxiety symptom networks during the first (June–July) and second (November–December) period of COVID-19 confinement in a population of older adults in the UK. This observation is of great relevance because the emotional disorders examined were identified as the most common conditions in response to the emergence of COVID-19 in that context^5^. In addition, this finding provided a deeper understanding of how items related to depression and anxiety were interconnected and how they were influenced by stressful events such as social confinement. These results were supported by the confirmation of invariance in the overall structures of both networks.

Network clusters indicate that during the first period of confinement by COVID-19, there was a greater number of symptoms within the anxiety community, and this because the "restless sleep (D3)" item of depression clustered with anxiety symptoms. Based on the above, two premises can be asserted, the first is that this symptom is associated with both depression and anxiety, as found in previous research^58^, and the second is that older adults in the UK who were evaluated reported restless sleep as an anxious symptomatology due to the stressful context of the COVID-19 pandemic. In addition, the clique-percolation method detected items from the anxiety group that were intertwined with the depression domain, which were: "Lack of control of worry (A2)", "Inability to relax (A4)", and "Irritability (A6)", as in the first state of social isolation by COVID-19, elderly individuals were exposed to greater anxious symptomatology due excessive worry and disorientation about the spread of viruses, the duration of social restrictions, or medical care^3,59^. In relation to the second network, only the item "Irritability (A6)" overlapped with the depression cluster, suggesting a reduced manifestation of anxious symptomatology in older adults and a possible more effective adaptation to social isolation due to COVID-19. This may be related to the use of technological tools to maintain interaction with family and friends, which may have contributed to greater emotional well-being in this group^60^. In addition, it was observed that this same item ("Irritability A6") was also present in the cross-lagged panel network model, which allowed us to infer that irritability was the only anxious symptom that remained stable during the two confinement periods.

In addition, items from the depression cluster were identified that overlapped with anxious symptomatology. It was observed that the symptoms of "Depressed mood (D1)" and "Unhappiness (D4)" were clustered within the anxiety community during the first confinement by COVID-19. These findings are consistent with the research of Kaiser et al.^61^, who also found that the depressive state item showed overlap with the anxiety domain in a group of psychiatric patients. Additionally, it is important to keep in mind that unhappiness is not an exclusive criterion of depression. For older adults with anxious symptomatology, they are likely to experience a sense of unhappiness due to constant worry about the possible consequences of the COVID-19 pandemic. These concerns may include health complications, lack of social interaction, and lifestyle changes, which contribute to their perception of unhappiness^62,63^. While in the second time of confinement by COVID-19, there was a greater number of overlapping items between both clusters, such as "Depressive mood (D1)", "Restless sleep (D3)", and "Anhedonia (D6)". Therefore, the presence of more pronounced comorbid symptoms with anxiety during this period may be attributed to the fact that older adults experienced greater uncertainty regarding the prolongation of social isolation during the second COVID-19 confinement, a situation like that experienced during the first quarantine^59,64^. It is important to recognize that the "Depressive mood (D1)" remains persistent in both evaluation periods and in the cross-lagged panel network model, which implies that it is a constant transdiagnostic symptom in both stages. In addition, this symptom has an overlapping presence in psychological distress (anxiety and depression) in the form of bridges, indicating an increased risk of connection with other symptoms in the network. This pattern is maintained in response to the effects of both COVID-19 quarantine periods. Additionally, in the cross-lagged panel network model having "Sleep problems (D3)" had the largest network effect toward nervousness.

After a review of the literature, only two studies have been found that use the Clique-Percolation methodology to learn about overlapping symptoms within the anxiety and depression communities^30,61^. These studies differ from our findings because they report a greater number of groupings, without any diagnostic justification, and refer to symptoms that do not belong to any symptomatological domain. While in the current study, the two symptomatological clusters of anxiety and depression are maintained, which are necessary to identify because, from a transdiagnostic perspective, there are symptoms and causes that are shared equally for both disorders^65,66^. This is important since excessive clustering of symptoms in different domains can lead to a lack of clarity in the representation of the two mental health diagnoses considered by the DSM-V, which are represented as the interaction of all their signs and symptoms together within a given period^67^. The results refer to greater precision and interpretation based on transdiagnostic models between two emotional disorders such as anxiety and depression^28,68^, given that the methodology used allows studying these mental health problems as systems^17,69,70^.

Network findings point out that common components (items) underlying both disorders of "depressed mood" and "irritability" may arise in the face of emotional processing deficits^71,72^. Specifically, these symptoms may be related to inadequate regulation of these emotions and other negative feelings such as fear and unhappiness^73,74^. Deficits in this regulatory process may be the main cause of transdiagnostic (overlapping) symptoms reinforcing each other 89,92, which may interact jointly with other symptoms in the network with greater intensity and duration, e.g., they may reinforce insomnia and worry that are linked to memory problems in older adults^75^. On the other hand, the cross-lagged panel network shows that the symptom of depressed mood is related to a greater degree of lack of control of worry, which is reinforced by a greater feeling of loneliness. In addition, it was observed that the item "Inability to relax (A4)" within the anxious symptomatology had a higher centrality (expected influence) in the networks during both periods of confinement by COVID-19. This symptom showed a strong connection with "Lack of control of worry (A2)", "Nervousness (A1)", and "Excessive worry (A3)".

These findings are in accordance with the predictability values, given that the first four anxiety items presented the highest degree of explained variance (R2); therefore, both centrality and predictability allowed us to understand which items are of greater relevance and relative importance in the interaction of symptoms within the depression and anxiety network. Such results may be explained by the fact that the pandemic and social isolation generated a distressing and uncertain environment in elderly individuals^76,77^, who were considered a population at risk for the increase in the number of COVID-19 infections and deaths^9^. Studies conducted in China during the first quarantine were reported, with similar findings highlighting the centrality of such anxiety symptoms in distress symptomatology network with young^46^, and adult participants^78^. This is also consistent with other research conducted in participants from Norway, the United States, the UK, and Australia^79,80^. Previous work only considered one period, while the current study evaluated two networks in two periods of COVID-19, where there was no significant difference between the item centrality values of both networks using the NCT^55^. Therefore, the inability to relax can be considered an essential symptom in the manifestation of anxiety and depression in older adults in the UK because of the impact of COVID-19.

Regarding depressive symptoms, it was identified that, in the first network, the item "Depressed mood (D1)" was the most central and influential, given that it maintained high intensity relationships with "Fatigue symptoms (D2)", "Unhappiness (D4)", and "Loneliness (D5)". During this period, COVID-19 mandatory confinement status had been initiated in multiple countries, including the UK, which facilitated isolation and social distancing in various households and families^1^. This loss of social contact came to increase feelings of sadness, loneliness, and depression in older adults due to limited visits and gatherings by friends and family^81^. This measure also presented a higher index of bridge expected influence, i.e., a high level of depressed mood may act as one of the nodes of greater connection between the symptomatological domains (anxiety and depression) of the network. This is in line with other research in patients with epilepsy who were attending a hospital outpatient clinic in China, where they found that the depressed mood item was the most central and bridging symptom within a network of anxiety and depression^82^.

In the second network the results were similar with respect to depressive symptoms, where "Depressed mood (D1)" had higher EI and BEI centrality, these two measures are used to understand the importance and impact of nodes in a network globally and on a cluster basis^48^. Therefore, it is likely that older adults who experience high levels of depressive feelings are more vulnerable to exhibit other symptoms of depression to a greater degree. One example is that depressed mood and fatigue may elicit more pronounced anxiety responses in this age group. This could be because, due to their age and state of health, it is common for them to experience both physical and emotional exhaustion. These factors could contribute to increased concern for the future and for their own health, intensifying anxiety responses^83,84^.

After outlining the overlap and centrality of the items, it is important to consider the strongest relationship between symptoms of both emotional disorders. Based on the above, the findings showed that the symptom of "Restless sleep (D3)" and "Inability to relax (A4)" were more related in the two confinement times by COVID-19. In studies published during the early period of the pandemic, positive relationships were found to exist with such symptoms in the anxiety and depression network in Chinese university students^85^, as well as in Norwegian university students^80^. In the second COVID-19 confinement, there were also studies showing that restless sleep was directly related to difficulty relaxing in health care professionals during the last stage of the pandemic (October 2020) in China^86,87^. This may be since the inability to relax alters the reconciliation of sleep, since it increases or reduces the hours of deep sleep, in addition, it produces a poor night's rest that generates greater feelings of tiredness during the day and a greater nervousness^88,89^.

In the cross-lagged panel network spanning the two pandemic waves, a clear pattern of influence directed toward the symptom "Nervousness (A1)" is observed. This influence comes mainly from the depressive symptom related to "Sleep problems (D3)", as well as other symptoms such as "Anhedonia (D6)" and "Hopelessness (D7)". These symptoms also exerted a direct effect on the symptom of nervousness in the network. This dynamic may be explained by the impact of the COVID-19 pandemic, which caused more pronounced difficulties in falling and staying asleep in older adults. This situation generated fatigue and exhaustion, which in turn increased the propensity to nervousness, due to the difficulties to effectively regulate emotions in this age group^90^. In addition, sleep problems are intensified when the individual experiences a loss of pleasure from performing previously enjoyable activities, as well as hopelessness about life^8^.

### Limitations

A limitation of this study lies in the fact that it addressed symptoms of depression and anxiety in older adults in the UK exclusively through surveys. Consequently, it would be important for future research to incorporate diagnostic evaluations to include clinical participants who present with specific mental problems, thereby broadening the understanding of these conditions in this population. Another limitation of this study lies in the analysis limited to the periods of confinement due to COVID-19 at the first and second moment. Therefore, it is essential to continue to investigate and examine these variables in subsequent periods, with the aim of strengthening the understanding of the stability of core and comorbid symptoms over time. Finally, it is important to note that few studies employ the Clique-Percolation approach in psychometric networks to represent clinical variables through clusters that share common underlying components. These models capture fundamental elements in transdiagnostic models of mental health, and their application could provide valuable insight into future research.

## Conclusion

In conclusion, this study evidences the presence of items that share manifestations between the depression and anxiety communities in older adults during the first and second confinement in the UK. In particular, "Irritability (A6)" and "Depressed mood (D1)" were identified as symptoms with prominent comorbidity. In addition, the depressive symptom exhibited a higher centrality of interconnectedness between clusters in both evaluation periods. In the cross-lagged panel network, it was observed that the “Nervousness symptom (A2)” was the most predictable measure in this structure. In addition, the item related to "Sleep problems (D3)" was identified as having a significant predictive impact, according to centrality indicators over time. These results also reaffirmed the presence of a transdiagnostic overlap between irritability and depressed mood.

### Supplementary Information

Supplementary Information.

## Data Availability

The data used in this study are available through the study website at https://www.elsa-project.ac.uk/ (accessed January 27, 2023).

## Abbreviations

COVID-19: Coronavirus disease 2019
SARS-CoV-2: Severe acute respiratory syndrome coronavirus 2
UK: United Kingdom
DSM-5: Diagnostic and Statistical Manual of Mental Disorders, 5th edition
CPM: Clique percolation method
EI: Expected influence
BEI: Bridge expected influence
CLPN: Cross-lagged panel network
EBIC: 'Extended Bayesian information criterion
