# Supplementary material for: Network analysis of anxiety and depressive symptoms during the COVID-19 pandemic in older adults in the United Kingdom
Source: Sci Rep. 2024 Apr 2;14:7741. doi: 10.1038/s41598-024-58256-8 (PMC10987576; doi:10.1038/s41598-024-58256-8)
Supplement: Supplementary file 1 — Supplementary Information. [file 41598_2024_58256_MOESM1_ESM.docx]

**APPENDICES**

**Appendix 1. Correlation Matrix Time 1.**

|  | A1 | A2 | A3 | A4 | A5 | A6 | A7 | D1 | D2 | D3 | D4 | D5 | D6 | D7 |
| --- | --- | --- | --- | --- | --- | --- | --- | --- | --- | --- | --- | --- | --- | --- |
| A1 | 0 |  |  |  |  |  |  |  |  |  |  |  |  |  |
| A2 | 0.259 | 0 |  |  |  |  |  |  |  |  |  |  |  |  |
| A3 | 0.26 | 0.284 | 0 |  |  |  |  |  |  |  |  |  |  |  |
| A4 | 0.163 | 0.095 | 0.124 | 0 |  |  |  |  |  |  |  |  |  |  |
| A5 | 0 | 0.061 | 0.048 | 0.293 | 0 |  |  |  |  |  |  |  |  |  |
| A6 | 0.114 | 0.001 | 0.083 | 0.115 | 0.102 | 0 |  |  |  |  |  |  |  |  |
| A7 | 0.171 | 0.151 | 0.154 | 0.062 | 0.072 | 0.075 | 0 |  |  |  |  |  |  |  |
| D1 | 0.015 | 0.069 | 0.02 | 0.042 | 0.04 | 0.056 | 0.031 | 0 |  |  |  |  |  |  |
| D2 | 0 | 0.029 | 0.039 | 0.032 | 0.024 | 0.057 | 0.003 | 0.221 | 0 |  |  |  |  |  |
| D3 | 0.02 | 0.003 | 0.029 | 0.14 | 0.065 | 0.054 | 0 | 0.038 | 0.012 | 0 |  |  |  |  |
| D4 | 0.032 | 0.038 | 0 | 0.06 | 0 | 0.015 | 0.015 | 0.138 | 0.013 | 0 | 0 |  |  |  |
| D5 | 0.011 | 0.032 | 0.023 | 0 | 0.041 | 0 | 0 | 0.13 | 0.039 | 0.007 | 0.115 | 0 |  |  |
| D6 | 0.026 | 0.031 | 0.015 | 0.023 | 0.006 | 0.027 | 0.019 | 0.094 | 0.05 | 0 | 0.444 | 0.079 | 0 |  |
| D7 | 0.015 | 0 | 0 | 0.016 | 0.012 | 0.035 | 0.021 | 0.039 | 0.359 | 0.106 | 0 | 0.096 | 0.114 | 0 |

**Note:** A1: Nervousness, A2: Worry management, A3: Worry, A4: Inability to relax, A5: Restlessness, A6: Irritable, A7: Fear, D1: Depressed, D2: Fatigue, D3: Restless sleep, D4: Happiness (R), D5: Loneliness, D6: Anhedonia (R), D7: Hopelessness.

**Appendix 2. Correlation Matrix Time 2.**

|  | A1 | A2 | A3 | A4 | A5 | A6 | A7 | D1 | D2 | D3 | D4 | D5 | D6 | D7 |
| --- | --- | --- | --- | --- | --- | --- | --- | --- | --- | --- | --- | --- | --- | --- |
| A1 | 0 |  |  |  |  |  |  |  |  |  |  |  |  |  |
| A2 | 0.256 | 0 |  |  |  |  |  |  |  |  |  |  |  |  |
| A3 | 0.247 | 0.324 | 0 |  |  |  |  |  |  |  |  |  |  |  |
| A4 | 0.155 | 0.097 | 0.133 | 0 |  |  |  |  |  |  |  |  |  |  |
| A5 | 0 | 0.064 | 0.042 | 0.286 | 0 |  |  |  |  |  |  |  |  |  |
| A6 | 0.116 | 0.019 | 0.081 | 0.121 | 0.087 | 0 |  |  |  |  |  |  |  |  |
| A7 | 0.146 | 0.143 | 0.127 | 0.058 | 0.117 | 0.064 | 0 |  |  |  |  |  |  |  |
| D1 | 0.045 | 0.066 | 0.027 | 0 | 0.028 | 0.063 | 0.048 | 0 |  |  |  |  |  |  |
| D2 | 0.004 | 0.014 | 0.014 | 0.034 | 0.031 | 0.049 | 0.062 | 0.188 | 0 |  |  |  |  |  |
| D3 | 0 | 0 | 0.05 | 0.149 | 0.059 | 0.046 | 0 | 0.053 | 0.03 | 0 |  |  |  |  |
| D4 | 0.034 | 0.011 | 0.013 | 0.047 | 0 | 0.035 | 0.042 | 0.147 | 0.024 | 0 | 0 |  |  |  |
| D5 | 0.016 | 0.028 | 0.026 | 0.016 | 0.033 | 0 | 0.016 | 0.152 | 0.051 | 0 | 0.076 | 0 |  |  |
| D6 | 0.032 | 0.028 | 0.015 | 0.035 | 0.017 | 0.009 | 0.021 | 0.064 | 0.062 | 0.009 | 0.402 | 0.101 | 0 |  |
| D7 | 0.014 | 0.036 | 0 | 0 | 0 | 0.063 | 0 | 0.048 | 0.383 | 0.09 | 0 | 0.054 | 0.111 | 0 |

**Note:** A1: Nervousness, A2: Worry management, A3: Worry, A4: Inability to relax, A5: Restlessness, A6: Irritable, A7: Fear, D1: Depressed, D2: Fatigue, D3: Restless sleep, D4: Happiness (R), D5: Loneliness, D6: Anhedonia (R), D7: Hopelessness.

**Appendix 3. Centrality measures for the CLPN model.**


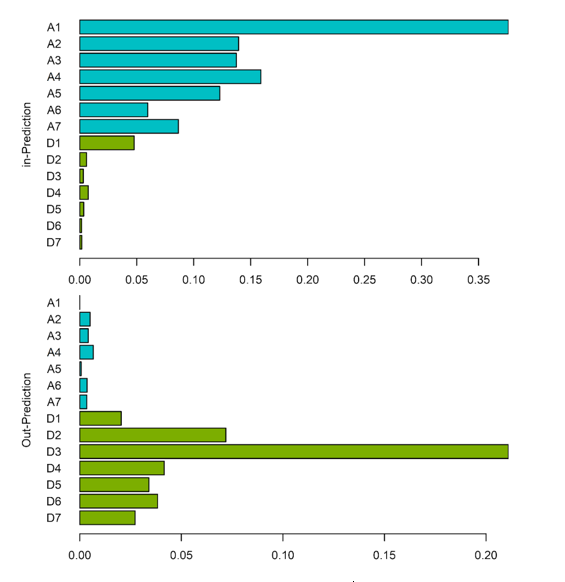


**Note:** A1: Nervousness, A2: Worry management, A3: Worry, A4: Inability to relax, A5: Restlessness, A6: Irritable, A7: Fear, D1: Depressed, D2: Fatigue, D3: Restless sleep, D4: Happiness (R), D5: Loneliness, D6: Anhedonia (R), D7: Hopelessness.

**Appendix 4. Matrix of standardized regressions of the CLPN model.**

|  | A1 | A2 | A3 | A4 | A5 | A6 | A7 | D1 | D2 | D3 | D4 | D5 | D6 | D7 |
| --- | --- | --- | --- | --- | --- | --- | --- | --- | --- | --- | --- | --- | --- | --- |
| A1 | 0 | 0 | 0 | 0 | 0 | 0 | 0 | 0 | 0 | 0 | 0 | 0 | 0 | 0 |
| A2 | 0 | 0.238 | 0 | 0 | 0 | 0 | 0 | 0 | 0 | 0 | 0 | 0 | 0 | 0 |
| A3 | 0 | 0 | 0.175 | 0 | 0 | 0 | 0 | 0 | 0 | 0 | 0 | 0 | 0 | 0 |
| A4 | 0 | 0 | 0 | 0.186 | 0 | 0 | 0 | 0 | 0 | 0 | 0 | 0 | 0 | 0 |
| A5 | 0 | 0 | 0 | 0 | 0.192 | 0 | 0 | 0 | 0 | 0 | 0 | 0 | 0 | 0 |
| A6 | 0 | 0 | 0 | 0 | 0 | 0.316 | 0 | 0 | 0 | 0 | 0 | 0 | 0 | 0 |
| A7 | 0 | 0 | 0 | 0 | 0 | 0 | 0.352 | 0 | 0 | 0 | 0 | 0 | 0 | 0 |
| D1 | 0 | 0.109 | 0 | 0 | 0 | 0 | 0 | 0.314 | 0 | 0 | 0 | 0 | 0 | 0 |
| D2 | 0 | 0 | 0.103 | 0.111 | 0 | 0 | 0.154 | 0 | 0.272 | 0 | 0 | 0 | 0 | 0 |
| D3 | 0.421 | 0 | 0 | 0 | 0 | 0 | 0 | 0 | 0 | 0.343 | 0 | 0 | 0 | 0 |
| D4 | 0 | 0 | 0 | 0.102 | 0.126 | 0 | 0 | 0 | 0 | 0 | 0.414 | 0 | 0 | 0 |
| D5 | 0 | 0.123 | 0 | 0 | 0 | 0 | 0 | 0 | 0 | 0 | 0 | 0.230 | 0 | 0 |
| D6 | 0.111 | 0 | 0 | 0.107 | 0 | 0 | 0 | 0 | 0 | 0 | 0 | 0 | 0.445 | 0 |
| D7 | 0.142 | 0 | 0 | 0 | 0 | 0 | 0 | 0 | 0 | 0 | 0 | 0 | 0 | 0.336 |

**Note:** A1: Nervousness, A2: Worry management, A3: Worry, A4: Inability to relax, A5: Restlessness, A6: Irritable, A7: Fear, D1: Depressed, D2: Fatigue, D3: Restless sleep, D4: Happiness (R), D5: Loneliness, D6: Anhedonia (R), D7: Hopelessness.

**Appendix 5.** *Mathematical analysis*

*Mathematical analysis*

The qgraph package allows accommodating the capabilities of different patterns through the visualization of networks, which consist of nodes (also called vertices), which are connected by axes that have a given weight, which indicates the strength of the connection and may or may not be directional^63^.

The axes can be represented by weight matrices, which indicate the connectivities between the different nodes in the form of a network matrix, this means that, for a graph with n nodes, its weight matrix A is an $n\times n$ square matrix, in which the element $a_{ij}$ represents the strength or weight of the connection from node i to node j, which can take any value, and that, if it is zero, it represents the absence of connection, while the strength of the connections is symmetric around zero, if the resulting graph is undirected and, in the opposite case, it would be directed; It can also happen that the weights are only 0 and 1, so it would be interpreted as an adjacency matrix and would be considered as a non-weighted graph ^64,65^.

This means that:

The matrix $x_{1}$ is symmetric:

$$x_{1}=\left[ \begin{matrix} 0 & 1 & 2 \\ 1 & 0 & 3 \\ 2 & 3 & 0 \end{matrix} \right]$$

And indicates that the connection between nodes 1 -- 2 and 2 - 1 is identical, as well as the connection of nodes 1 - 3 and 3 - 1; as well as nodes 2 - 3 and 3 - 2.

While the $x_{2}$ matrix is asymmetric:

$$x_{2}=\left[ \begin{matrix} 0 & 1 & 2 \\ 0 & 0 & 3 \\ 0 & 0 & 0 \end{matrix} \right]$$

And indicates that there is only connection between nodes 1 - 2; 1 - 3 and 2 - 3, only in that exclusive direction.

Finally, the matrices $x_{3}$ and $x_{4}$ are the unweighted matrix representation for the matrices presented above:

$x_{3}=\left[ \begin{matrix} 0 & 1 & 1 \\ 1 & 0 & 1 \\ 1 & 1 & 0 \end{matrix} \right]$ $x_{4}=\left[ \begin{matrix} 0 & 1 & 1 \\ 0 & 0 & 1 \\ 0 & 0 & 0 \end{matrix} \right]$

This makes it possible that different metrics can be used as axis weights, e.g., correlations, covariances, regression parameters, factor loadings, log likelihoods, which can be easily computed.

The most typical thing to find in the practice of psychometric network analysis are partial correlations, which, if the variables to be analyzed are continuous and have normal distributions, can be obtained from the inversion of the correlation matrix (or covariance); in this case, if p is the inverse of the correlation matrix, the partial correlation $w_{ij}$ of variables i and j can be calculated as follows:

$$w_{\mathrm{ij}}=\frac{-p_{\mathrm{ij}}}{\sqrt{p_{\mathrm{ii}}p_{\mathrm{jj}}}}$$

However, in the field of psychology, we usually encounter small databases, which have been solved by the application of the 'absolute minimum reduction and selection operator' (LASSO) as a form of penalized regularization; by limiting the total sum of the absolute values of the parameters, which causes many axis weights to reduce to exactly zero and drop out of the model, resulting in a highly interpretable sparse or conservative network, This can be used in combination with the 'extended Bayesian information criterion' (EBIC) for model selection, both Ising and GGM^44^, also called the Glasso operator.

It is also possible to use a transformation on the data for undirected graphs, known as 'non-paranormal', to smooth the normality assumption needed in high dimensionality problems, which applies an error penalty of type $l_{1}$ with non-paranormal regularization to the GLASSO model, based on non-parametric marginal ^66^.

It is also possible to use a selection model other than EBIC, such as ric, which starts with the assumption of generating data by rotation that have the same probability as the original data. This implies that, for any orthogonal matrix Q, the error distribution does not change with rotations, leading to an error distribution that depends only on its magnitude, known as a spherically symmetric distribution. From this, permutations are performed to ensure model accuracy^52^, which has been shown to be more beneficial in empirical terms^49^.

After performing the partial correlation network analysis with the different penalties, the next step is to use the bootnet package to perform a second stage, which involves analyzing the network structure to infer the central nodes.

In this specific context, the expected influence was used as a metric to identify the central nodes, regardless of whether they were related to the presence of the clusters. A more exhaustive description of this metric is available in the corresponding section of the method.

From this first reasoning, the expected influence as well as the bridging expected influence can be applied in two steps^57^, where the first step of the expected influence (IE1) can be calculated as follows:

$$IE_{1_{i}}=\sum_{j=1}^{N} a_{ij}w_{ij}$$

Where $IE_{1_{i}}$ is the sum of the weights of the axes shared between i with the other j nodes in the network; $a_{ij}$ is an adjacency matrix with binary elements where 1 indicates presence and 0 absence between nodes ij; finally, $w_{ij}$ is also an adjacency matrix with ranges between -1 and 1 indicating the weight of the axis between nodes ij. This implies that if all links in the network are positive, the anticipated influence in the first step will be equal to the measure of strength. However, if there are negative links, the expected influence will tend to be less than the strength ^67^.

The second step of the expected influence is represented by the following equation:

$$IE_{2_{i}}=\sum_{j=1}^{N} a_{ij}w_{ij}+\sum_{j=1}^{N} a_{ij}w_{ij}\sum_{k=1}^{N} a_{jk}w_{jk}$$

In this case, we build on the basis of the first step to add that, the $IE_{2}$ of a node i is its $IE_{1}$ plus the product of $IE_{1}$ with $\sum_{k=1}^{N} a_{jk}w_{jk}$, where $a_{jk}w_{jk}$ denotes the weight of the axes between node j and all other nodes in the network [k]. This means that the secondary influence between nodes ij will vary depending on the strength of their axis.

For the case of centrality in the form of bridging expected influence, this can be represented by the following equation:

$$bridging expected influence = \sum_{b\in\left( N\left( a \right)-C \right)} w_{ab}$$

Where C represents a set of nodes in a community within the network; in this will define each measure of bridging centrality with respect to node a, where $a\in C$. N(a) is used to denote the set of nodes adjacent to node a. $w_{ab}$ represents the weight of each axis ab in the network. The variations for each step of the expected natural influence are applied to this formula ^68^.

As a next step, the accuracy of the parameters and measurements of the found network system is estimated. This is done through the Bootstrap technique in two types: non-parametric and case-dropping, applied for each weight of the axes and the centralities respectively ^69^.

On the other hand, communities within a network system can be explored in a variety of ways, among the most common are the walktrap^70^ and Louvain algorithms, with its more robust derivative Leiden^71^. However, there are also other methods, which for psychometric networks are still being evaluated, but which from the beginning have accurate results, such as the spinglass algorithm^55^.

The inherent limitation of these algorithms lies in their inability to identify whether certain nodes overlap in two or more communities simultaneously. The membership of these algorithms only allows a node "i" to belong to a single community at a time, which is established by its modularity measure ^72^. To correct this fact of exclusive membership, which cannot be admissible in complex node networks, as is the case of symptoms in psychometric networks, the superposition of communities through the method of clique percolation was devised ^73^.

This method is based on the CFinder algorithm, which in turn was designed under the conception of the clique percolation algorithm, originally for unweighted networks (Palla et al., 2005), which was later derived in weighted networks^74^.

This algorithm acts based on the number of k-cliques that each node a has in connection with other nodes “bc” in a network system, this assumes that the minimum number of allowed cliques is $k = 3$. Based on this idea, we have established a community as a set of adjacent cliques sharing precisely $k-1$ nodes. To illustrate, if we consider a prior value of $k=3$, we can state that 2 cliques of 3 nodes are adjacent if they share exactly 2 nodes, which is equivalent to the presence of an axis between them.

In a network without weights, let us consider a first set of cliques: a-b-c and a second set of cliques: a-b-d. These two sets can form a community since they share the same nodes a-b. Now, if we add more nodes to the example, with cliques such as d-e-f, d-e-g, d-f-g, and e-f-g, which in turn form a new community, we will notice that these two communities share a common node: d. This node becomes what is called a "percolated" or "overlapping" node, since it is present in both communities at the same time.

In the context of weighted networks, the algorithm requires performing an intermediate step to determine whether the intensity (calculated as the geometric mean of the axis weights) of the cliques exceeds the set threshold, denoted as I.

This value is defined by the following formula:

$$I_{C}=\left( \prod_{i<j; i,j\backslash inC} w_{ij} \right)^{2/k\left( k-1 \right)}$$

Where C is the clique, ij are the nodes, w is the weight of the axis between those nodes and k is the size of the clique.

This practice can be combined with entropy and stability measurements for the overlying communities, in addition to observing the modularity of the communities and their degree of influence on the percolated nodes.

Ultimately, among the practices that are applied when there are two networks in which we want to establish a relationship to observe their differences, is the technique of comparing networks based on permutations ^75^.

The NCT (NetworkComparisonTest) algorithm performs a three-step process to make comparisons: 1) It analyzes the observed data, estimates the networks and calculates the corresponding statistical values; 2) It generates a reference distribution by resampling the original data, estimates the network structures for each iteration and calculates the statistics; 3) Evaluates the significance of contrasting the network obtained in the first stage with the reference distribution generated in the second stage.

For the difference between networks, 3 aspects are analyzed: 1) the invariant network structure; 2) the invariant strength of the axes; 3) the global invariant strength; which distributes the differences into global and local.

The evaluation of the invariant network structure is performed based on the null hypothesis that postulates that all axes are equal $H_{0}:\Omega^{1}=\Omega^{2}$. In other words, if any difference is found in the axes of the second network compared to the first one, it is concluded that the networks are distinct. However, carrying out this test in a comprehensive manner proves to be a laborious process. To address this issue, the resampling-based permutation technique is used. Using this approach, all values of the axis weights are tested using the maximum statistic, with the objective of ensuring rigorous control of the familywise range of error (FWER), allowing the significance of the α-value to be fully determined from this process.

This difference in networks between the single axes of each can be defined by this maximum statistic for $i<j$ as:

$$M\left( \omega^{1},\omega^{2} \right)=\max_{ij} \left| \omega_{ij}^{1}-\omega_{ij}^{2} \right|$$

Since this assumption assumes that both networks share identical axes, any significant discrepancy may appear minimal in the data, especially if we consider the variation inherent in sampling. In this context, the following identity is presented:

$$x:maxf\left( x \right)<d=Unionx:f\left( x \right)>d$$

In this sense, we can state that when the maximum value of all differences exceeds a predefined threshold "d", which indicates statistical significance, then at least one of the other differences must also exceed this same threshold "d". Equivalently, if the maximum value of all differences does not reach statistical significance, then none of the other differences will be significant in this context.

In relation to the constant shaft force, this is determined under consideration of the same null hypothesis $H_{0}:\omega_{ij}^{1}=\omega_{ij}^{2}$, however, focuses on the variations of the absolute ratios between the specific axis forces $\omega_{ij}$ at nodes i and j $i<j$) of networks 1 and 2:

$$E\left( \omega_{ij}^{1},\omega_{ij}^{2} \right)=\left| \omega_{ij}^{1}-\omega ij^{2} \right|$$

For this procedure, different control corrections for the FWER error can be used, such as the Bonferroni-holm correction, which is recommended for improving the performance of the significance coefficient α^60^.

Finally, there is the global invariant force, in which the assumption is made that connectivity levels are the same across subpopulations. This overall connectivity can be treated as global strength, which is obtained by summing all the absolute values of the axes in the network. For $i<j$, the distance S between two networks is defined by the following equation:

$$S\left( \omega^{1},\omega^{2} \right)=\left| \sum_{i=1}^{p} \sum_{j>i} \left( \left| \omega_{ij}^{1} \right|-\left| \omega_{ij}^{2} \right| \right) \right|$$

In this way, the null hypothesis is calculated in the population:

$$H_{0}:\sum_{i=1}^{p} \sum_{j>i} \left| \omega_{\mathrm{ij}}^{1} \right|=\sum_{i=1}^{p} \sum_{j>i} \left| \omega_{\mathrm{ij}}^{2} \right|$$

This analysis acquires meaning and relevance when there is a certainty or a suspicion, even in an exploratory approach, that the nodes in one network are more densely interconnected than in the other, or if one network presents different conditions than the other. The lack of difference at this level does not necessarily imply that the two networks are similar. For this reason, it is important to also consider the invariance of the network. It may be the case that the value of S is not significant, but the value of M indicates that at least one axis is different, and vice versa.

Among the temporal analyses that can be applied to the network perspective is the CLPN perspective, a method that evaluates the relationships between individual items over time. It is applied in two steps: 1) It fits a series of regularized regression models to estimate the temporal cross-lag and autoregression coefficients; 2) Summarize the results with graphs and compute summary statistics such as in-prediction and out-prediction^62^.

To calculate the regression coefficients of each variable in the second period (T2), both in relation to itself and to the other variables in the first period (T1), the following formula is used:

$$x_{j.T2}=\beta_{j}^{'}x_{T1}+\varepsilon_{j}$$

Where $x_{j.T2}$ is the variable j in T2; $\beta_{0}$ is the intercept; $\beta_{j}$ is the regression coefficient corresponding to the prediction of the vector $x_{T1}$, which contains all p variables in T1; $\varepsilon_{j}$ is the residual. These regression coefficients are calculated with a maximum likelihood penalty and lasso penalty^76^. This compresses all small regressions to be exactly zero, while making the other paths longer. The result obtained is a sparse network, in which many routes from T1 to T2 will be estimated as exactly zero.

Regularized regression estimates are calculated from:

$$\frac{1}{N}\sum_{i=1}^{N} l\left( x_{i,j,T2},\hat{\beta}_{0,j}+\hat{\beta}_{j}^{'}x_{i,T1} \right)+\lambda_{j}\parallel\hat{\beta_{j}}\parallel_{1}$$

Where i = 1...N denotes the individual. This minimizes the sum of the individual likelihoods plus a penalty; $\lambda_{j}\parallel\hat{\beta_{j}}\parallel_{1}$ in which, $\parallel\hat{\beta_{j}}\parallel_{1}$ is the sum of the absolute values of the coefficients $\hat{\beta_{j}}$. Finally, $\lambda_{j}$ is a tuning parameter that determines the strength of the penalty. Regression estimates are obtained from a sequence of 100$\lambda$ and, the one that produces the lowest cross-validation error is selected.

After this, we proceed to the calculation of the in-prediction and out-prediction of the estimated network, represented as centrality measures. The first (in-prediction) examines a variable in the second period (T2) that is predicted by the other variables in the first period (T1). This calculation involves determining the proportion of the variability present in each variable at T2 that can be explained by the full set of variables at T1. The resulting values of this ratio can vary between 0 and 1. The formula to calculate it is as follows:

$$\mathrm{inPre}d_{j}=\frac{\mathrm{var}\left( \hat{x}_{j,T2} \right)}{\mathrm{var}\left( \hat{x}_{j,T2} \right)}=\frac{\mathrm{var}\left( \hat{\beta}_{0,j}+\hat{\beta}_{j}^{'}x_{i,T1} \right)}{\mathrm{var}\left( \hat{x}_{j,T2} \right)}$$

On the other hand, the second measure (out-prediction) can be understood as the ability of each variable in the first period (T1) to predict the other variables in the second period (T2). Its calculation involves the sum of the squares of the standardized regression coefficients going from the target variable at T1 to each of the variables at T2. This measure is represented by the following formula:

$$outPred_{j}=\sum_{k=1}^{p} \hat{\beta}_{x_{k,}T1x_{j,}T2}^{2}$$
